# Thermal evolution offsets the elevated toxicity of a contaminant under warming: A resurrection study in *Daphnia magna*

**DOI:** 10.1111/eva.12637

**Published:** 2018-05-07

**Authors:** Chao Zhang, Mieke Jansen, Luc De Meester, Robby Stoks

**Affiliations:** ^1^ Evolutionary Stress Ecology and Ecotoxicology KU Leuven Leuven Belgium; ^2^ Laboratory of Aquatic Ecology, Evolution and Conservation KU Leuven Leuven Belgium

**Keywords:** ecotoxicology, evolution, global warming, nanoparticles, resurrection ecology, risk assessment, synergism, thermal tolerance

## Abstract

Synergistic interactions between temperature and contaminants are a major challenge for ecological risk assessment, especially under global warming. While thermal evolution may increase the ability to deal with warming, it is unknown whether it may also affect the ability to deal with the many contaminants that are more toxic at higher temperatures. We investigated how evolution of genetic adaptation to warming affected the interactions between warming and a novel stressor: zinc oxide nanoparticles (nZnO) in a natural population of *Daphnia magna* using resurrection ecology. We hatched resting eggs from two *D. magna* subpopulations (old: 1955–1965, recent: 1995–2005) from the sediment of a lake that experienced an increase in average temperature and in recurrence of heat waves but was never exposed to industrial waste. In the old “ancestral” subpopulation, exposure to a sublethal concentration of nZnO decreased the intrinsic growth rate, metabolic activity, and energy reserves at 24°C but not at 20°C, indicating a synergism between warming and nZnO. In contrast, these synergistic effects disappeared in the recent “derived” subpopulation that evolved a lower sensitivity to nZnO at 24°C, which indicates that thermal evolution could offset the elevated toxicity of nZnO under warming. This evolution of reduced sensitivity to nZnO under warming could not be explained by changes in the total internal zinc accumulation but was partially associated with the evolution of the expression of a key metal detoxification gene under warming. Our results suggest that the increased sensitivity to the sublethal concentration of nZnO under the predicted 4°C warming by the end of this century may be counteracted by thermal evolution in this *D. magna* population. Our results illustrate the importance of evolution to warming in shaping the responses to another anthropogenic stressor, here a contaminant. More general, genetic adaptation to an environmental stressor may ensure that synergistic effects between contaminants and this environmental stressor will not be present anymore.

## 1 INTRODUCTION

Global warming and environmental pollution are two global stressors which, moreover, may interact with each other synergistically. Indeed, many studies showed an increased toxicity of contaminants at higher temperatures (Dinh et al., 2013; Moe et al., 2013; Salafsky et al., 2008; Sokolova & Lannig, 2008). For example, the review by Holmstrup et al. (2010) revealed ca. 80% of the studies showed a synergistic interaction between a contaminant and heat stress for traits such as survival, developmental rate, and hatching success. Jackson, Loewen, Vinebrooke, and Chimimba (2016) showed that ca. 30% of the studies testing combined effects of warming and contaminants in freshwater ecosystems showed a synergism for traits such as growth and reproduction. Hence, whether organisms can locally survive under warming depends not only on their ability to cope with the increasing temperature, but also on their ability to deal with the elevated toxicity of contaminants at higher temperatures. To understand the impact of both warming (Stoks, Geerts, & De Meester, 2014) and contaminants (Brady, Richardson, & Kunz, 2017), an evolutionary perspective is important. This is because the strong selection imposed by these stressors can lead to rapid evolutionary changes (Hendry, 2016). Rapid evolution increasing the tolerance to warming (e.g., Geerts et al., 2015; Ljungfeldt, Quintela, Besnier, Nilsen, & Glover, 2017) and to contaminants (e.g., Brady et al., 2017; Turko, Sigg, Hollender, & Spaak, 2016) has indeed been reported. Yet, despite the increasing concern for interactions between warming and contaminants, we do not know how rapid evolution in response to warming changes the toxicity of contaminants at higher temperatures.

The emerging pattern is that when an environmental stressor that challenges an organism to expend energy (such as warming‐induced stress) is combined with a toxicant, this results in a synergism between both stressors (Liess, Foit, Knillmann, Schäfer, & Liess, 2016). We hypothesize that evolution to deal with one stressor will reduce and may even offset the synergistic interaction with a second stressor. This can be expected because when a population evolves tolerance to a certain stressor that environmental condition is no longer experienced as a stressor (Vam Straalen, 2003). With regard to warming, the acquisition of genetic adaptation to higher temperatures has been demonstrated in several taxa and has been linked to changes in gene expressions (e.g., Garvin, Thorgaard, & Narum, 2015; Gleason & Burton, 2015; Narum, Campbell, Meyer, Miller, & Hardy, 2013; Porcelli, Butlin, Gaston, Joly, & Snook, 2015; for the study species: Jansen et al., 2017; Yampolsky et al., 2014). Thermal evolution is expected to reduce the energetic costs of dealing with warming, thus leaving more energy to deal with stressors such as toxicants, thereby potentially offsetting the synergism between the toxicant and warming.

One powerful approach to investigate evolution is resurrection ecology (Franks, Hamann, & Weis, 2018; Weider, Jeyasingh, & Frisch, 2018). This approach reconstructs microevolution by comparing within a population the phenotypes of organisms hatched from ancestral eggs with those from descendants hatched from more recent eggs (Orsini et al., 2013). Resurrection ecology has been successfully applied to document evolutionary responses of natural populations to warming (e.g., Cuenca Cambronero, Zeis, & Orsini, 2018; Geerts et al., 2015) and to contaminants (e.g., Kuester, Wilson, Chang, & Baucom, 2016; Turko et al., 2016). In contrast, this approach has been used only rarely to investigate the consequences of evolution in response to one stressor for a population's ability to deal with a second stressor (but see Zhang, Jansen, De Meester, & Stoks, 2016).

A particular type of “contaminants of emerging concern” that is getting increased attention are nanoparticles (Blinova, Ivask, Heinlaan, Mortimer, & Kahru, 2010; Ma, Williams, & Diamond, 2013). Engineered nanoparticles are novel anthropogenic stressors whose levels are expected to rise because of incremental consumer use (Jarvis, Miller, Lenihan, & Bielmyer, 2013). While an increasing number of studies looked at the effects of contaminants under warming (Carrie et al., 2009; Moe et al., 2013; Sokolova & Lannig, 2008), surprisingly few have focused on nanocontaminants (but see Mos, Kaposi, Rose, Kelaher, & Dworjanyn, 2017; Wong & Leung, 2014). Zinc oxide nanoparticles (nZnO) are one of the most produced nanoparticles with a worldwide production estimated at 550 tons per year (Piccinno, Gottschalk, Seeger, & Nowack, 2012; Read et al., 2016). Accordingly, they have been detected in the environment at concentrations of up to 17.1 mg per kg dry mass in sludge (Read et al., 2016). There is increasing concern of their impact in aquatic environments due to the local accumulation and the fact that most aquatic species cannot escape exposure (Spisni, Seo, Joo, & Su, 2016).

We examined whether evolution to warming changes the ability to deal with the novel contaminant nZnO at higher temperatures using resurrection ecology in the water flea *Daphnia magna*. *Daphnia* are important organisms to address evolutionary responses to stressors as they are keystone species in aquatic food webs (Miner, De Meester, Pfrender, Lampert, & Hairston, 2012) and model species in ecotoxicology (OECD 2004). Moreover, as *Daphnia* produce resting eggs that are viable for many years, one can reconstruct evolution across decades (Orsini et al., 2013). We hatched dormant eggs of two subpopulations (old: 1955–1965, recent: 1995–2005) of *D. magna* from a well‐characterized lake in UK (Sayer, Davidson, Jones, & Langdon, 2010). This lake has experienced a steady increase in average temperature and in the frequency and intensity of heat waves over time (Geerts et al., 2015) and has never been exposed to contaminants (Sayer, Burgess et al., 2010). In this *Daphnia* population, animals from the recent “derived” subpopulation evolved a higher extreme heat tolerance (measured as upper thermal temperature where animals become immobile) than those from the old “ancestral” subpopulation (Geerts et al., 2015). This makes an ideal system to look how rapid evolution to one stressor (here warming) changes the ability to deal with a second, novel stressor (here nZnO), and particularly how the tolerance to nZnO at higher temperatures evolved in response to thermal evolution.

Given the widespread occurrence of synergistic interactions between warming and metals (Moe et al., 2013; Sokolova & Lannig, 2008), we predicted stronger toxic effects of nZnO under warming in the old subpopulation. Given that thermal evolution makes warming less stressful, we expected the recent subpopulation to show a smaller (or no) increase in sensitivity to nZnO under warming. We tested these predictions for intrinsic growth rate, a key fitness‐related trait. We predicted nZnO exposure to reduce intrinsic growth rate especially under warming in the old subpopulation, reflecting a synergism, and that this stronger negative effect of nZnO under warming will be less pronounced or absent in the recent subpopulation. To obtain insights in the underlying mechanisms, we also tested for effects on physiology and the internal bioaccumulation of zinc. Specifically, we measured metabolic activity through quantifying the RNA:DNA ratio (Pauwels, Stoks, & De Meester, 2010) and quantified a key metal detoxification mechanism (metallothionein genes, Amiard, Amiard‐Triquet, Barka, Pellerin, & Rainbow, 2006). As defense against contaminants is expected to be energetically costly, we also measured the major energy storage molecules (fat, sugars, and proteins).

## 2 MATERIALS AND METHODS

### 2.1 Study system

*D. magna* dormant eggs were obtained from the layered egg bank of Felbrigg Hall Lake (52°54.10′N, 1°15.19′E, North Norfolk, UK), which is situated in an old estate run by the National Trust and has never been exposed to industrial waste (Sayer, Burgess et al., 2010). *D. magna* has been continuously present in this lake since the 1940s (Davidson, Sayer, Langdon, Burgess, & Jackson, 2010; Sayer, Burgess et al., 2010). Dormant eggs were isolated from two sediment layers corresponding to 1955–1965 and 1995–2005, hereafter called the old and recent periods. The recent period experienced an increase in average temperature of 1.2°C and a threefold increase in heat waves compared to the old period (Met Office 2012). Paleolimnological data indicate that the level of eutrophication and macrophyte coverage were highly similar in these two periods (Sayer, Davidson et al., 2010). X‐ray fluorescence analysis showed the contamination levels (including Zn) in both periods were low and similar (mean levels of Zn in the sediment, 1955–1965: 125.9 ± 2.0 μg/g; 1995–2005: 102.2 ± 1.7 μg/g) (C. Sayer & T. Davidson, unpublished data). Given that genetic data using 43 microsatellite markers demonstrated genetic continuity between clones from the old and recent period (Geerts et al., 2015), we consider clones from both periods as belonging to two subpopulations of the same *D. magna* population (hereafter called old and recent subpopulation).

Subpopulations of *D. magna* were resurrected from resting eggs and kept in monoclonal cultures for several years under standard laboratory conditions (20°C, photoperiod of 14:10 L:D). Among the hatched clones, which were all genetically distinct, seven clones were randomly selected from each subpopulation, resulting in a total of 14 clonal lineages. The clones used here are a subset from the clones used in the study by Geerts et al. (2015). Before the exposure experiment, they were cultured in the test medium under standard conditions (20°C, photoperiod of 14:10 L:D, fed 1 × 10^5^ cells/ml *S. obliquus* daily, medium refreshed every other day) for seven generations (around 3 months). This minimized interference from maternal effects and allowed acclimation to the test medium. During this period, *Daphnia* were reared in ISO 6341 medium (CaCl_2_•2H_2_O: 0.294 g/L, MgSO_4_•H_2_O: 0.123 g/L, NaHCO_3_: 0.065 g/L, KCl: 0.006 g/L) that is recommended for toxicity testing of metals (OECD 2004). Each generation was started from juveniles of the second clutch.

### 2.2 Experimental setup

To test the effects of warming and nZnO on life history and physiology, we ran a full factorial experiment where all clones of both subpopulations were exposed to each combination of two temperatures (20 and 24°C) and two nZnO treatments (nZnO absent and present). We chose 20°C as it is the approximate average summer water temperature in waterbodies inhabited by the study species in UK (Van Doorslaer et al., 2010). The higher temperature of 24°C simulates the predicted 4°C increase in global average surface temperature by 2100 under IPCC (2013) scenario RCP8.5. Based on a range‐finding experiment of all 14 clones, we obtained an EC_50,48 hr_ immobilization of 860 μg/L Zn for neonates. We used a nominal concentration of 86 μg/L (10% of the EC_50,48 hr_) as sublethal concentration for the chronic exposure in the actual experiment. This nZnO concentration is within the range of previously reported chronic EC_50_ values for *D. magna* reproduction (Adam, Leroux, Knapen, Bals, & Blust, 2015) and is also environmentally relevant. For example, the estimated nZnO concentrations in UK waterbodies go up to 100 μg/L (Boxall, Tiede, & Chaudhry, 2007). As we were not interested in clonal differences but in differences between both periods, clones were used as replicates at the subpopulation level (Van Doorslaer et al., 2010).

The mothers of the experimental *Daphnia* were reared in 500 ml vials filled with 450 ml ISO 6341 medium at 20°C and 24°C for one generation (Zhang et al., 2016). Per clone, 40 neonates from the second brood were reared at the same temperature as the mother generation and randomly divided between the two nZnO treatments. As a result, one cohort of 20 *Daphnia* from a given clone was reared together in a 500‐ml vial filled with 450 ml medium at each of the four treatment combinations of temperature and nZnO. *D. magna* were fed daily with 1 × 10^5^ cells/ml *S. obliquus,* and the culture medium was refreshed every other day. The measured zinc concentrations (at the moment the medium was freshly renewed, mean ± *SD*,* n *=* *5 pooled samples) were 72.01 ± 2.14 μg/L at 20°C and 73.24 ± 2.45 μg/L at 24°C as verified with inductively coupled plasma mass spectrometry (Agilent 7700× ICP‐MS, Biocompare, USA). The associated water quality parameters (mean ± *SD*,* n *=* *5 samples) across groups were the following: pH: 7.99 ± 0.03, conductivity: 623.23 ± 1.55 μS/cm, dissolved oxygen: 8.99 ± 0.19 mg/L, hardness: 239.10 ± 2.75 mg/L CaCO_3_.

The stock solution of nZnO was prepared using commercial nano zinc oxide dispersion (nanoparticles with average size < 35 nm, pH 7.0 ± 0.1; Sigma‐Aldrich, Louis, MO, USA). The nanoparticles were characterized using a Zeiss EM900 transmission electron microscope (Carl Zeiss, Oberkochen, Germany) at a concentration of 5 mg/L (Figure S1). An initial stock solution of 5 × 10^3^ mg/L Zn was prepared by ultrasonification of nZnO in an ultrasonic bath (Elmasonic S40, Elma®, Germany) for 30 min in MilliQ water. The solution was then stored at 4°C under darkness. Each time a test solution was made, the stock solution was ultrasonicated for 30 min at maximum power to eliminate aggregates.

### 2.3 Response variables

For each cohort of 20 individuals, hence per vial, we quantified the intrinsic growth rate (“*r*”). The intrinsic growth rate was calculated based on the data of the first two broods using the Euler equation (Roff, 2001): 1 = ∫*e* ^‐*rx*^ *l*_*x*_ *m*_*x*_dx. Here, *l*_*x*_ represents the proportion of survivors at age *x* and *m*_*x*_ represents the number of offspring released at day *x*. To interpret any effects on “*r*,” we also quantified age at maturity (defined as the time at which half of the animals in a given vial carried brood in their brood pouch) and number of offspring (counted every day for the first two broods). The number of offspring that was released by the cohort was counted and removed every day to keep densities in each vial constant throughout the experiment. To keep the manuscript focused, these life‐history subcomponents are reported in Appendix S2. After the release of their second brood, *D. magna* samples from a given vial were flash‐frozen in liquid nitrogen and stored in three separate sets at −80°C for further analyses. The animals in the cohorts developed under each treatment combination sufficiently synchronously so that we could stop vials when all animals had released their second clutch and while there were no visual signs of the third clutch in the brood pouches.

One set of ten frozen *Daphnia* per vial was pooled for measuring the physiological responses. *Daphnia* were first homogenized in 150 μl MilliQ water (giving a total volume of ca. 200 μl). The homogenate was then split into three parts: (i) 40 μl was weighed by drying at 60°C for 24 hr using preweighed tin capsules to get the dry body mass; (ii) 120 μl was used to measure body Zn concentrations using inductively coupled plasma mass spectrometry (Agilent 7700x ICP‐MS, Biocompare, USA); and (iii) another 40 μl was analyzed for the quantification of protein, fat, glycogen, and glucose contents using a microplate reader (Infinite M200, TECAN, Switzerland) following established protocols for the study species (Zhang et al., 2016). The homogenate was first diluted twice in MilliQ water and centrifuged for 5 min at 13,000 *g* under 4°C. To measure the fat content, we mixed 8 μl of the supernatant with 56 μl sulfuric acid (100%) in acetone rinsed glass tubes and heated for 20 min at 150°C and added 64 μl of MilliQ water thereafter. We filled wells of a 384 well microliter plate with 30 μl of this mixture and measured the absorbance at 340 nm. Absorbances were converted to fat contents based on a standard curve of glyceryl tripalmitate. For total sugar content (glucose and glycogen), 25 μl supernatant, 65 μl MilliQ water, and 10 μl amyloglucosidase (Sigma A7420, 1 unit/10 μl) were mixed together in wells of a 96‐well microliter plate and incubated for half an hour at 37°C, which allowed transformation of glycogen to glucose. Subsequently, we added 200 μl of glucose assay reagent (Sigma G3293) to each well and measured the glucose. Then, we incubated the mixture at 30°C for another 20 min and measured the absorbance at 340 nm. Absorbances were converted into glucose contents based on a standard curve of glucose. To measure the protein content, we used the protocol described by Bradford (1976). Briefly, 1 μl homogenate was diluted in 160 μl MilliQ water, 40 μl of Bio‐Rad Protein Dye reagent concentrate was added, and the whole was mixed vigorously. The absorbance at 595 nm was measured after 5 min at 37°C. Absorbance was converted into protein contents based on a standard curve of bovine serum albumin. Samples were run in duplicate (glucose and glycogen) or triplicate (fat and protein), and the means per sample were used for the statistical analyses. Patterns for glucose and total sugar contents (glucose + glycogen) were very similar; therefore, only the total sugar content was reported. Protein, fat, and total sugar contents and the Zn burdens were expressed as μg per mg dry mass.

One *Daphnia* per vial was used for quantifying the RNA:DNA ratio based on Vrede, Persson, and Aronsen (2002). RNA:DNA is a good proxy for metabolic activity and relies on the principle that the total RNA content is primarily a function of ribosome number (hence increasing with higher production of proteins), whereas DNA content remains constant in an individual (Pauwels et al., 2010). *Daphnia* were homogenized in an extraction buffer (50 mM EDTA, 0.05% SDS in 50 mM Tris), and 100 μl homogenate and 2 μl EB (ethidium bromide, 100 μg/ml) were mixed and incubated for 15 min on ice. The total amount of RNA + DNA was quantified at an excitation/emission wavelength of 535:595 nm. Next, the RNA was broken down by adding 1 μl of RNAse solution (20 mg/ml) to another 100 μl aliquot of the sample and incubated for 1 hr at room temperature; then, the DNA was measured in the same way. RNA and DNA concentrations were measured in triplicate, and their mean ratios per sample were used in the statistical analyses.

One set of four frozen *Daphnia* per vial was pooled to quantify gene expression levels of two metallothionein genes (MT‐a, MT‐b) that play a role in metal detoxification in *Daphnia* (Shaw et al., 2007). Primer sequences and the qRT–PCR protocol were from Poynton et al. (2008). Briefly, total RNA was isolated using the Trizol extraction method (Invitrogen, Belgium) following DNase treatment (Fermentas, Germany). The purity and concentration of RNA were measured with a NanoDrop ND‐1000 spectrophotometer (NanoDrop Technologies). A standardized 300 ng of extracted RNA was reverse‐transcribed with QuantiTect Reverse Transcription Kit (Qiagen). 18S rRNA was assayed to normalize for total cDNA in each sample. PCR amplification was performed using a SYBR Green Master Mix with an ABI Prism 7000 Sequence Detection System (Applied Biosystems, Foster City, CA) at the following conditions: 2 min at 95°C and 40 cycles each consisting of 15 s at 95°C and 1 min at 60°C. The melting curve was included to verify amplification specificity. RT–PCR data were analyzed using GenEx software (version 6, MultiD) for quality control, normalization, transformation, and gene expression‐level analysis.

### 2.4 Statistical analyses

To test for effects of temperature, nZnO exposure, and subpopulation on the response variables, three‐way ANOVAs were performed. As we measured one cohort of each clone under each of the four treatment combinations, clone nested in subpopulation was added as a random factor. To more clearly show the different patterns between the two subpopulations, two‐way ANOVAs were conducted separately for each subpopulation. Significance levels were not corrected for multiple testing (Moran, 2003). Of the 56 *p*‐values (across 8 ANOVAs, Table 1), one would expect to erroneously assign significance in 2.8 cases by chance alone (assuming α = 0.05). All tests were run in the GLM module of STATISTICA v12.0 (Stat‐soft, Tulsa, OK, USA). Except for the internal zinc concentrations, which were log‐transformed, the assumptions of ANOVA (normally distributed errors and homogeneity of variances) were met for all variables. When an interaction effect between temperature and the nZnO treatment was detected, the type of interaction was identified by calculating the interaction effect size (estimated as Hedges'd with 95% confidence interval) (Jackson et al., 2016). An interaction effect size larger than zero indicates a synergistic interaction among stressors, while an interaction effect size smaller than zero indicates an antagonistic or reversal interaction. The latter occurs when the observed combined effect is opposite to the predicted combined additive effect.

**Table 1:** Summary of ANOVAs testing for the effects of temperature, nZnO and subpopulation on life history and physiology of *D. magna*

	*df*	Temperature (T)		nZnO (Zn)		Subpopulation (S)		T × Zn		T × S		Zn × S		T × Zn × S	
		*F*	*p*	*F*	*p*	*F*	*p*	*F*	*p*	*F*	*p*	*F*	*p*	*F*	*p*
Zn concentration	1, 36	0.00	.956	147.13	**<.0001**	1.49	.230	36.98	**<.0001**	0.69	.411	13.36	**<.01**	4.86	**<.05**
Intrinsic growth rate	1, 36	0.40	.532	24.78	**<.0001**	0.04	.845	3.19	.083	11.17	**<.01**	0.52	.474	3.75	.061
RNA:DNA	1, 36	28.01	**<.0001**	2.63	.114	0.84	.364	12.84	**<.01**	5.12	**<.05**	10.20	**<.01**	8.29	**<.05**
Sugar content	1, 36	4.24	**<.05**	5.11	**<.05**	3.62	.065	4.18	**<.05**	1.09	.302	0.42	.523	0.39	.537
Fat content	1, 36	1.95	.171	3.65	.064	0.20	.657	4.40	**<.05**	0.07	.786	0.00	.997	0.07	.791
Protein content	1, 36	1.97	.169	0.44	.513	4.62	**<.05**	0.37	.548	7.73	**<.05**	0.71	.403	4.91	**<.05**
MT‐a	1, 36	0.89	.767	0.68	.415	0.12	.736	1.80	.188	2.91	.097	0.66	.423	2.01	.165
MT‐b	1, 36	0.42	.522	41.10	**<.0001**	0.12	.729	6.11	**<.05**	1.11	.293	1.76	.193	1.37	.249

Significant *p*‐values are marked in bold. Significance levels are not corrected for multiple testing. Of the 56 *p*‐values (across eight ANOVAs), one would expect to erroneously assign significance in 2.8 cases by chance alone (assuming α = 0.05).

## 3 RESULTS

### 3.1 Internal zinc concentrations

**Figure 1:**
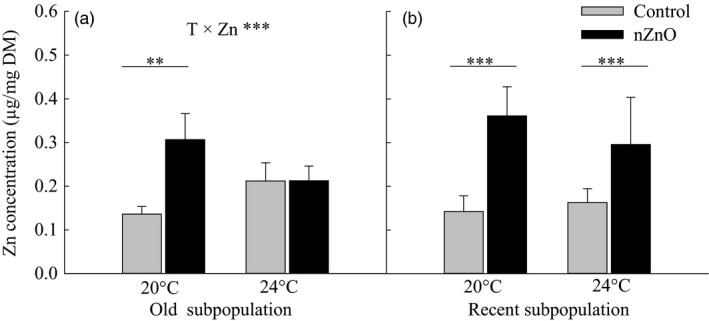
Body Zn concentrations as a function of nZnO exposure and rearing temperature in the old and recent subpopulations of *Daphnia magna* from Felbrigg Hall Lake. Given are least‐squares means + 1 *SE* based on seven clones per subpopulation. The asterisks indicate significant differences between the nZnO treatments levels within a given rearing temperature (*p < .05, **p < .01, ***p < .001). Significant Temperature × nZnO interactions are indicated per subpopulation based on separate two‐way ANOVAs

No *Daphnia* died during the exposure period. *Daphnia* exposed to nZnO had higher Zn burdens in their bodies (*F*_1,36_ = 147.13, *p *<* *.0001), except for those of the old subpopulation at 24°C, resulting in a significant Temperature × nZnO × Subpopulation interaction (*F*_1,36_ = 4.86, *p *<* *.05, Table 1, Figure 1).

### 3.2 Life history

**Figure 2:**
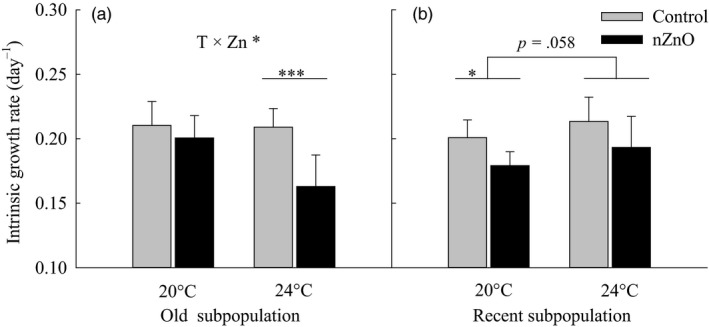
Intrinsic growth rate as a function of nZnO and temperature in the old and recent subpopulations of *Daphnia magna* from Felbrigg Hall Lake. Given are least‐squares means + 1 *SE* based on seven clones per subpopulation. The asterisks indicate significant differences between the nZnO treatments levels within a given rearing temperature (*p < .05, **p < .01, ***p < .001). Significant Temperature × nZnO interactions are indicated per subpopulation based on separate two‐way ANOVAs

*Daphnia* had lower intrinsic growth rates when exposed to nZnO (Table 1, Figure 2). There was a trend (*p *=* *.061) for a Temperature × nZnO × Subpopulation interaction for intrinsic growth rate (Table 1, Figure 2). Separate two‐way ANOVAs showed indeed that the Temperature × nZnO interaction differed between subpopulations. In the old subpopulation, nZnO strongly reduced the intrinsic growth rate at 24°C (−22.1%) but not significantly at 20°C (−4.6%), indicating a synergism (Temperature × nZnO, *F*_1,24_ = 6.43, *p *=* *.018; Hedges'd = 0.26, 95% CI: [0.02; 0.49], Table S2, Figure 2a). Instead, in the recent subpopulation, there was no such synergism, but rather a similar moderate reduction in intrinsic growth rate under nZnO exposure at both temperatures (20°C: −10.8%, 24°C: −9.4%) (main effect nZnO, *F*_1,24_ = 9.77, *p *<* *.01; Temperature × nZnO, *F*_1,24_ = 0.01, *p *=* *.913, Figure 2b).

From the perspective of warming, these different Temperature × nZnO interactions between subpopulations reflect a pattern of thermal evolution. The significant Temperature × nZnO interaction in the old subpopulation indicated that warming did not affect intrinsic growth rate in the control and reduced intrinsic growth rate in the presence of nZnO (Figure 2a). The absence of the Temperature × nZnO interaction in the recent subpopulation indicated that warming in general (across both nZnO levels) tended to increase intrinsic growth rate (main effect Temperature *p *=* *.058, Figure 2b).

### 3.3 Physiology

**Figure 3:**
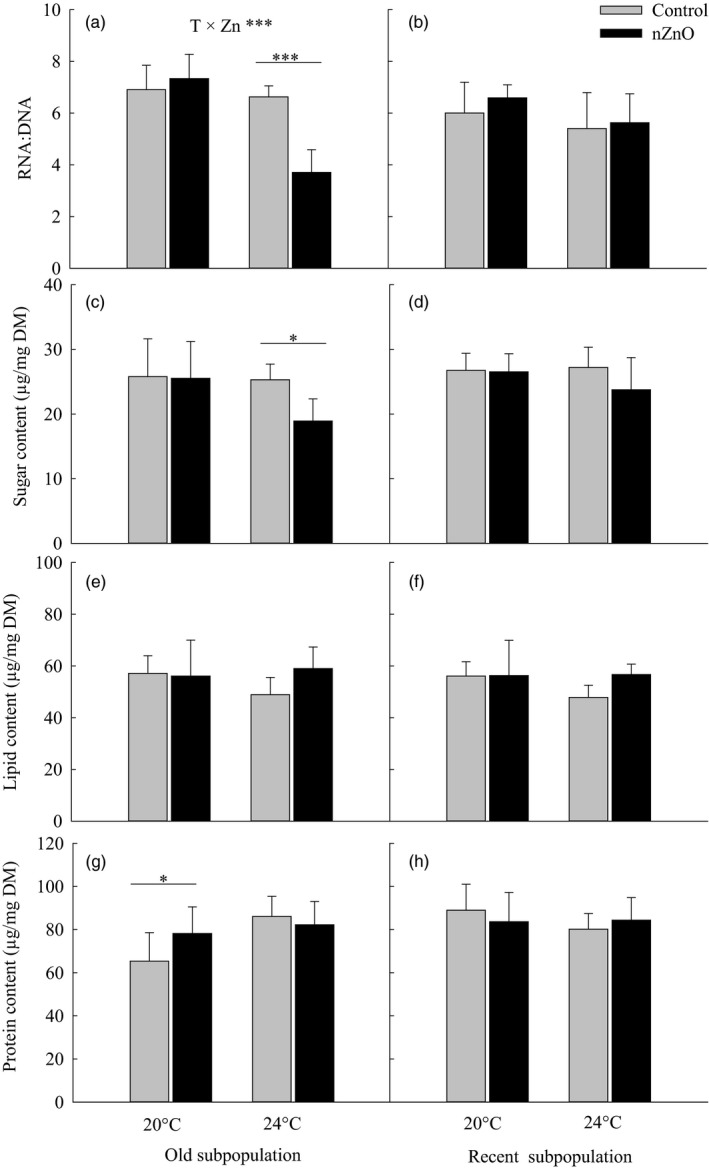
RNA:DNA ratio (a,b), sugar content (c,d), lipid content (e,f), and protein content (g,h) as a function of nZnO and temperature in the old and recent subpopulations of *Daphnia magna* from Felbrigg Hall Lake. Given are least‐squares means + 1 *SE* based on seven clones per subpopulation. The asterisks indicate significant differences between the nZnO treatments levels within a given rearing temperature (*p < .05, **p < .01, ***p < .001). Significant Temperature × nZnO interactions are indicated per subpopulation based on separate two‐way ANOVAs

In the old subpopulation, exposure to nZnO decreased the RNA:DNA ratio at 24°C but not at 20°C indicating a synergism (Hedges'd = 3.72, 95% CI: [3.03; 4.41], Table S2, Figure 3a); this synergism was not observed in the recent subpopulation (Temperature × nZnO × Subpopulation, *p *<* *.01, Table 1; Temperature × nZnO in the old subpopulation: *F*_1,24_ = 28.84, *p *<* *.0001, in the recent subpopulation: *F*_1,24_ = 0.19, *p *=* *.665, Figure 3a,b).

The effects of warming and nZnO strongly differed between the three energy storage molecules. For both subpopulations, nZnO only decreased the sugar content at 24°C (Temperature × nZnO; synergistic interaction: Hedges'd = 2.30, 95% CI: [1.40; 3.19], Table S2, Figure 3c,d), but the decrease in the old subpopulation (−25.2%) was almost twice that of the recent subpopulation (−12.8%). In both subpopulations, warming decreased the fat content only when not exposed to nZnO (Temperature × nZnO; antagonistic interaction: Hedges'd = −3.33, 95% CI: [−4.38; −2.28], Table S2, Figure 3e,f). Only in the old subpopulation, warming increased the protein content when *Daphnia* were not exposed to nZnO, while neither temperature nor nZnO affected the protein content in the recent subpopulation (Table 1, Figure 3g,h).

**Figure 4:**
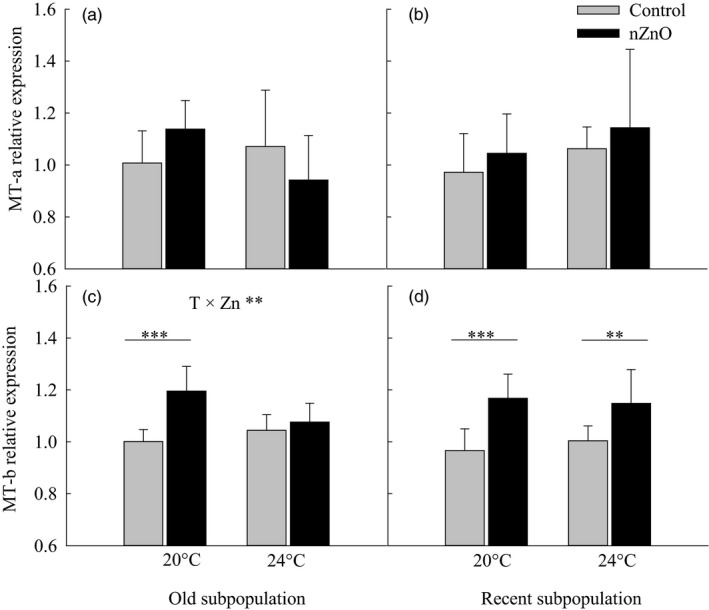
Relative expression levels of the metallothionein genes MT‐a (a,b) and MT‐b (c,d) as a function of nZnO and temperature in the old and recent subpopulations of *Daphnia magna* from Felbrigg Hall Lake. Given are least‐squares means + 1 *SE* based on seven clones per subpopulation. The asterisks indicate significant differences between the nZnO treatments levels within a given rearing temperature (*p < .05, **p < .01, ***p < .001). Significant Temperature × nZnO interactions are indicated per subpopulation based on separate two‐way ANOVAs

In general, nZnO increased the expression of MT‐b but not of MT‐a (Table 1, Figure 4). Separate ANOVAs showed in the old subpopulation that nZnO increased the MT‐b expression at 20°C but not at 24°C (Temperature × nZnO, *F*_1,24_ = 7.92, *p *=* *.0096, Figure 4c), indicating an antagonistic interaction (Hedges'd = −0.54, 95% CI: [−0.89; −0.19], Table S2). In the recent subpopulation, nZnO consistently increased MT‐b expression at both temperatures (nZnO, *F*_1,24_ = 19.90, *p *=* *.0002; Temperature × nZnO, *F*_1,24_ = 0.56, *p *=* *.461, Figure 4d).

## 4 DISCUSSION

Using resurrection ecology, we provide empirical evidence that thermal evolution can change the interactions between warming and a contaminant in a keystone species in freshwater ecosystems. The key finding was that the increased toxicity of this nanocontaminant under warming only occurred in the old subpopulation but not in the recent subpopulation. In other words, we demonstrated that thermal evolution could change the synergistic interaction between a contaminant and warming.

### 4.1 A higher toxicity of nZnO under warming in the old subpopulation

Warming did not affect the intrinsic growth rate in the old subpopulation when nZnO was absent. This was because of opposing effects on its subcomponents: *D. magna* matured earlier, yet suffered a lower fecundity at 24°C than at 20°C (Appendix S2). Previous studies in *D. magna* also showed an earlier maturation and a decreased fecundity under 4°C warming (Van Doorslaer et al., 2010; Zhang et al., 2016), but in these studies, the effect on maturation prevailed, resulting in higher intrinsic growth rates under warming.

While exposure to nZnO increased the internal Zn burden at 20°C, this only resulted in a decreased fecundity while no other measured response variables were affected. This may be partly explained by the upregulation of the detoxification gene metallothionein MT‐b under nZnO exposure at 20°C. The related MT‐a gene was not induced by nZnO in this study, which is similar to previous findings showing that in *D. magna,* MT‐b is more sensitive to metal exposure compared to MT‐a (Poynton et al., 2008).

Consistent with the synergism between contaminants and warming, exposure to nZnO was only toxic under warming. This became evident not only by lower levels of the measured fitness trait (intrinsic growth rate), but also in the lower RNA:DNA ratio and lower sugar content when *Daphnia* were exposed to nZnO at 24°C than at 20°C. A higher toxicity of trace metals under warming has been documented in the study species (Kimberly & Salice, 2014) as well as in other aquatic invertebrates (Debecker, Dinh, & Stoks, 2017; Dinh et al., 2013). In contrast, interaction effects between warming and nanocontaminants have been rarely studied. Wong and Leung (2014) reported interactive effects of warming and nZnO in three different marine organisms, and 4°C warming exacerbated the effects of nZnO on the larvae of the sea urchin *Tripneustes gratilla* (Mos et al., 2017). Both studies focused on marine organisms, and our study extended this by showing a keystone species in fresh water also had an increased sensitivity to a nanocontaminant under warming.

Given that the synergism occurred without an increase in total internal zinc burden at 24°C, an enhanced net uptake rate did not underlie the synergism. Instead, a changed accumulation of nZnO in specific target tissues or cells may have affected metabolic processes (De Schamphelaere et al., 2004). The decreased RNA:DNA ratio under nZnO exposure at 24°C suggesting a lower metabolic activity may have contributed to the observed life‐history shifts under higher temperatures, resulting in a lower intrinsic growth rate. The lower sugar content further adds that the energy storage was reduced under nZnO at 24°C, indicating less energy was available to defend against the nZnO and repair damage. That the nZnO only affected the sugar content and not the fat and protein contents matches another study on *D. magna* showing that the sugar content was more sensitive to zinc compared to protein and fat contents (Muyssen, Janssen, & Bossuyt, 2002). We hypothesize that a suppressed food intake, a common response to metal exposure both in *Daphnia* (Lari, Gauthier, Mohaddes, & Pyle, 2017; Zhu, Chang, & Chen, 2010) and other species (Hoseini, Rajabiesterabadi, & Kordrostami, 2016; Janssens, Dinh, Debecker, Bervoets, & Stoks, 2014), may have driven the reduced energy storage and contributed to the higher toxicity of nZnO at 24°C. Accordingly, as *D. magna* can accumulate a considerable amount of zinc from its food (Memmert, 1987), a suppressed food intake may also explain the absence of a higher internal zinc burden at 24°C. Finally, animals may have suffered a lower defense to nZnO under warming because of increased metabolic costs associated with higher temperatures; this mechanism was demonstrated for *D. magna* exposed to cadmium within a similar temperature range (Heugens et al., 2003). In support of this, the metallothionein detoxification gene MT‐b was upregulated under nZnO exposure at 20°C but not at 24°C.

Nano zinc can dissolve rapidly but also aggregate when it enters the aquatic environment (Adam et al., 2014). Whether the nanoparticles or the dissolved ions are responsible for the toxicity depends on the concentration of the nanoparticle suspensions. In our experiment, the exposure concentration was very low and the toxic effects under lower concentrations are commonly attributed to the dissolved ions (Li, Lin, & Zhu, 2013; Mos et al., 2017), and this was explicitly demonstrated by Xiao, Vijver, Chen, and Peijnenburg (2015). Also, the small difference in temperature (4°C) has been shown to have limited influence on dissolution rates of nZnO (Mos et al., 2017).

### 4.2 Thermal evolution resulted in no higher toxicity of nZnO under warming

The recent subpopulation, which was separated by 40 years from the old subpopulation, could better deal with warming, indicating thermal evolution. Indeed, compared to the old subpopulation, the recent subpopulation showed a less strong negative effect of warming on fecundity and a stronger positive effect of warming on accelerating maturation (see Appendix S2). Together, this resulted only in the recent subpopulation in a trend for a positive effect of warming on intrinsic growth rate. This matches the observation of rapid evolution of increased tolerance to endure extreme warm temperatures in the same *D. magna* population (Geerts et al., 2015). Two aspects of the thermal regime changed between both periods in the study region (Met Office 2012): an increase in average temperature with 1.2°C and a threefold increase in the number of heat waves (average temperature >30°C for two or more consecutive days). These may have been the driving forces for the thermal evolution both in terms of the life‐history response to 4°C warming (this study) and in terms of the increased ability to deal with extreme warm temperatures (Geerts et al., 2015). Given that *D. magna* normally shows one sexual generation per year, this evolution occurred across ca. 40 sexual generations (Fisk, Latta, Knapp, & Pfrender, 2007). In another *D. magna* population, thermal evolution associated with a strong heat wave changed life history over an even shorter time interval (ca. 7 years) (Zhang et al., 2016).

The key finding was that thermal evolution resulted in the disappearance of the synergistic effect between nZnO and warming. In the old subpopulation, nZnO decreased the intrinsic growth rate and the RNA:DNA ratio at 24°C but not at 20°C, while these synergistic interactions disappeared in the recent subpopulation. Similarly, although nZnO decreased the sugar content at 24°C in both subpopulations, the decline in the recent subpopulation was only half of that in the old subpopulation. Noteworthy, in terms of the intrinsic growth rate, the recent subpopulation not only evolved to be less sensitive to nZnO at 24°C but also evolved to be more sensitive to nZnO at 20°C. This may reflect a trade‐off where adaptation to higher temperatures comes at the cost of adaptation to lower temperatures. Both opposing evolutionary changes in sensitivity to nZnO may have contributed to the disappearance of the synergism.

This is the first demonstration that thermal evolution may change the widespread synergism where contaminants get more toxic at higher temperatures (Holmstrup et al., 2010; Noyes & Lema, 2015). This resembles the long‐term evolutionary pattern where cold‐adapted high‐latitude populations of the damselfly *I. elegans* are more sensitive to zinc at 24°C compared to 20°C (hence a synergism), while warm‐adapted low‐latitude populations are not (Dinh et al., 2013). Synergisms between contaminants and environmental variables are expected when the environmental variable itself imposes stress (Liess et al., 2016). By making exposure to 24°C no longer stressful, thermal evolution may therefore explain why the nZnO was no longer more toxic at 24°C than at 20°C. In contrast with the *Daphnia* from the old subpopulation, *Daphnia* from the recent subpopulation did not decrease their metabolic activity and did not suffer reduced energy storage in the presence of nZnO at 24°C. This may explain why only the *Daphnia* from the recent subpopulation were able to mobilize energy to defense at 24°C as shown by the elevated expression of the metallothionein gene MT‐b.

Felbrigg Lake, from where the tested *D. magna* population was isolated, is situated in an old estate run by the National Trust and has never been exposed to industrial waste (Sayer, Burgess et al., 2010). Both periods had low concentrations of zinc and the recent period had an even lower concentration compared to the old period (see Materials and methods), which indicates that the here observed evolution impacting the interaction between warming and nZnO cannot be explained by an increase in Zn pollution. Although we cannot completely exclude the possibility that other variables may differ between the two periods, it seems highly likely that thermal differences were the main reason causing the observed evolutionary changes, particularly because the responses to nZnO were temperature‐dependent.

### 4.3 Evolutionary perspectives for ecological risk assessment under global warming

It remains challenging to understand and predict the impact of contaminants under warming (Moe et al., 2013; Noyes et al., 2009). In the old subpopulation, we identified for this nanocontaminant the well‐known pattern in trace metals of a higher toxicity at higher temperatures. This suggests that in the absence of evolution, the predicted temperature increase of 4°C in 2100 under IPCC (2013) scenario RCP8.5 might increase the susceptibility toward the used sublethal nZnO concentration in this population. Instead, the resurrection approach demonstrated that thermal evolution might result in no higher toxicity of nZnO under warming. This novel insight highlights the importance of considering in situ thermal evolution when assessing ecological risks of contaminants in a warming world (Malaj et al., 2014). More general, besides warming, many other environmental stressors may magnify the toxicity of contaminants (Holmstrup et al., 2010; Liess et al., 2016). This generates the hypothesis that evolution leading to genetic adaptation to an environmental stressor may ensure that synergistic effects between contaminants and this environmental stressor will not be present anymore. While we provided proof of principle of this idea, more empirical tests will be needed under more experimental conditions (contaminant concentrations and temperatures) and for more species to explore the generality of our results. An extra dimension in this context may be that exposure to environmental stressors (being biotic or abiotic) may slow the speed of evolution to the contaminant (Becker & Liess, 2015). These all indicate that the increasing interest for multistressor effects in both fundamental and applied ecology (Côté, Darling, & Brown, 2016) would benefit from an explicit evolutionary perspective.

## CONFLICT OF INTEREST

We declare no conflict of interest.

## DATA ARCHIVING STATEMENT

Data for this study are available from the Dryad Digital Repository: https://doi.org/doi:10.5061/dryad.kh6jg77
