# Supplementary material for: Thermal evolution offsets the elevated toxicity of a contaminant under warming: A resurrection study in Daphnia magna
Source: Evol Appl. 2018 May 7;11(8):1425–36. doi: 10.1111/eva.12637 (PMC6099814; doi:10.1111/eva.12637)
Supplement: Supplementary file 1 [file EVA-11-1425-s001.docx]

Supporting information

**Thermal evolution offsets the elevated toxicity of a contaminant under warming: a resurrection study in *Daphnia magna***

**Appendix S1** - Characterization of the nZnO

**Appendix S2** – Life history results

**Appendix S3** - Results of the effect sizes

Number of pages: 5

Number of tables: 2

Number of figures: 2

**Appendix S1** - Characterization of the nZnO


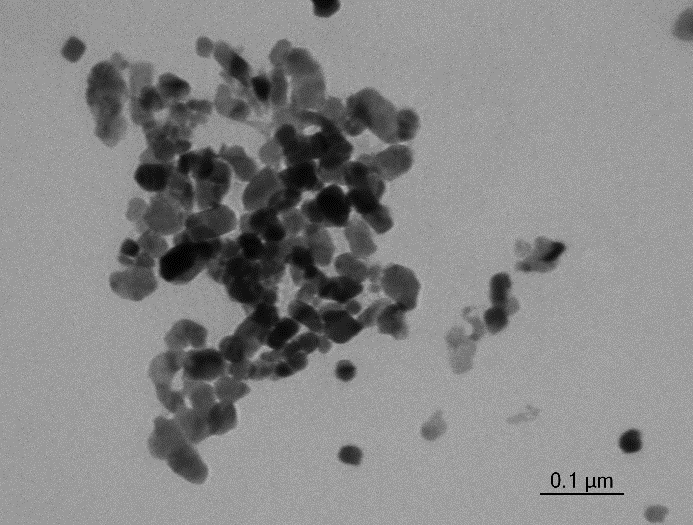


**Figure S1** TEM image of nZnO in the ISO 6341 medium, showing most nanoparticles are spherical with sizes between 20 and 50 nm.

**Appendix S2** - Life history results

*Daphnia* matured earlier and had a lower fecundity at the higher temperature, while they had a lower fecundity when exposed to nZnO (Table S1, Figure S2). Notably, the temperature effect on life history variables differed between subpopulations (Temperature × Subpopulation, Table S1, Figure S2). The recent *Daphnia* subpopulation dealt better with warming: the positive effect of warming on earlier maturation was more pronounced (27.6% compared to 15.3%) while the negative effect on fecundity was smaller (-35.3% compared to -50.5%) in the recent subpopulation compared to the old subpopulation (Table S1, Figure S2).

**Table S1**. Summary of ANOVAs testing for the effects of temperature, nZnO and subpopulation on age at maturity and fecundity of *D. magna*. Significant *P*-values are marked in bold.

|  | Age at maturity | | Fecundity | |
| --- | --- | --- | --- | --- |
| Effects | *F_1, 36_* | *P* | *F_1, 36_* | *P* |
| Temperature (T) | 114.72 | **< 0.0001** | 108.98 | **< 0.0001** |
| nZnO (Zn) | 3.47 | 0.07 | 22.78 | **< 0.0001** |
| Subpopulation (S) | 0.00 | 1.00 | 1.27 | 0.27 |
| T × Zn | 0.00 | 1.00 | 1.29 | 0.26 |
| T × S | 7.81 | **< 0.01** | 6.91 | **< 0.05** |
| Zn × S | 0.22 | 0.64 | 0.35 | 0.56 |
| T × Zn × S | 0.22 | 0.64 | 0.78 | 0.38 |

**Figure S2** Age at maturity (a, b) and fecundity (number of juveniles in the first two broods) (c, d) as a function of nZnO and temperature in the old and recent subpopulations of *Daphnia magna* from Felbrigg Hall Lake. Given are least-squares means + 1 SE based on seven clones per subpopulation. The asterisks indicate significant differences between rearing temperatures across the nZnO treatments (* *P* < 0.05, ** *P* < 0.01, *** *P* < 0.001). Significant Temperature × nZnO interactions are indicated per subpopulation based on separate two-way ANOVAs.

**Appendix S3**- Results of the effect sizes

**Table S2** Effect sizes for the temperature and nZnO exposure and their interactions.

| Subpopulations | Response | Interaction effect size  Hedges' d | Variances for  interaction effect size | Standard deviation for interaction | Lower 95% CI | Upper 95% CI | Interaction type | Effect size of T  Hedges' d | Variance for T individuals effect size | Effect size of Zn Hedges' d | Variance for Zn individuals effect size |
| --- | --- | --- | --- | --- | --- | --- | --- | --- | --- | --- | --- |
| both | sugar | 2.2958 | 0.1699 | 1.7061 | 1.4021 | 3.1895 | Synergism | -0.0057 | 0.1429 | -0.0548 | 0.1429 |
| both | fat | -3.3296 | 0.2391 | 2.0130 | -4.3841 | -2.2751 | Antagonism | -1.4367 | 0.1797 | -0.0320 | 0.1429 |
| old subpop. | "r" | 0.2566 | 0.2159 | 0.3177 | 0.0213 | 0.4919 | Synergism | -0.0808 | 0.2859 | -0.5234 | 0.2955 |
| old subpop. | RNA:DNA | 3.7167 | 0.5432 | 0.9299 | 3.0278 | 4.4056 | Synergism | -0.6175 | 0.2993 | 0.4257 | 0.2922 |
| old subpop. | MT-b | -0.5435 | 0.2213 | 0.4698 | -0.8916 | -0.1954 | Antagonism | 0.6170 | 0.2993 | 1.7788 | 0.3987 |
